# XBP-1s Promotes B Cell Pathogenicity in Chronic GVHD by Restraining the Activity of Regulated IRE-1α-Dependent Decay

**DOI:** 10.3389/fimmu.2021.705484

**Published:** 2021-10-01

**Authors:** Hee-Jin Choi, Chih-Hang Anthony Tang, Linlu Tian, Yongxia Wu, M. Hanief Sofi, Taylor Ticer, Steven D. Schutt, Chih-Chi Andrew Hu, Xue-Zhong Yu

**Affiliations:** ^1^ Microbiology & Immunology, Medical University of South Carolina, Charleston, SC, United States; ^2^ Center for Translational Research in Hematologic Malignancies, Houston Methodist Cancer Center, Houston Methodist Research Institute, Houston, TX, United States; ^3^ Hollings Cancer Center, Medical University of South Carolina, Charleston, SC, United States

**Keywords:** allo-HCT, chronic GVHD, IRE-1α, XBP-1, RIDD, ER stress, UPR

## Abstract

Allogeneic hematopoietic cell transplantation (allo-HCT) is an effective therapeutic procedure to treat hematological malignancies. However, the benefit of allo-HCT is limited by a major complication, chronic graft-versus-host disease (cGVHD). Since transmembrane and secretory proteins are generated and modified in the endoplasmic reticulum (ER), the ER stress response is of great importance to secretory cells including B cells. By using conditional knock-out (KO) of XBP-1, IRE-1α or both specifically on B cells, we demonstrated that the IRE-1α/XBP-1 pathway, one of the major ER stress response mediators, plays a critical role in B cell pathogenicity on the induction of cGVHD in murine models of allo-HCT. Endoribonuclease activity of IRE-1α activates XBP-1 signaling by converting unspliced XBP-1 (XBP-1u) mRNA into spliced XBP-1 (XBP-1s) mRNA but also cleaves other ER-associated mRNAs through regulated IRE-1α-dependent decay (RIDD). Further, ablation of XBP-1s production leads to unleashed activation of RIDD. Therefore, we hypothesized that RIDD plays an important role in B cells during cGVHD development. In this study, we found that the reduced pathogenicity of XBP-1 deficient B cells in cGVHD was reversed by RIDD restriction in IRE-1α kinase domain KO mice. Restraining RIDD activity per se in B cells resulted in an increased severity of cGVHD. Besides, inhibition of RIDD activity compromised B cell differentiation and led to dysregulated expression of MHC II and costimulatory molecules such as CD86, CD40, and ICOSL in B cells. Furthermore, restraining the RIDD activity without affecting XBP-1 splicing increased B cell ability to induce cGVHD after allo-HCT. These results suggest that RIDD is an important mediator for reducing cGVHD pathogenesis through targeting XBP-1s.

## Introduction

Although it is an effective therapy to treat hematological disease, the benefit of allogeneic hematopoietic stem cell transplantation (allo-HCT) is limited by the induction of complications such as acute and chronic graft-versus-host disease (GVHD) (1, 2). Chronic GVHD (cGVHD) is still the main cause for the morbidity and mortality of long-term survivors after allo-HCT (3). Despite the continuous effort to understand and reduce the cGVHD pathogenicity, the practical option for treatment is still very limited except for steroids. Therefore, new strategies to both prevent and treat cGVHD are urgently required.

It is well established that T cells play a major role in GVHD development, but emerging evidence from pre-clinical studies and clinical trials emphasize the importance of B cell involvement in the pathology of cGVHD (4–6). B cells from cGVHD patients showed increased B cell receptor response and resistance to apoptosis resulting in the constant activation of B cells (5, 7, 8). The presence of auto-antibody secreting B cells promoted by alloreactive donor CD4 T cells is an important mediator of autoimmune and fibrotic features of cGVHD (4, 9, 10). Very recently, the US Food and Drug Administration approved ibrutinib, a Bruton’s tyrosine kinase inhibitor, which is important for B cell receptor signaling, as second-line therapy of steroid-refractory cGVHD (11). It shows that B cell regulation can be one of the essential targets for cGVHD treatment. Thus, we focus on the role of the unfolded protein response (UPR) mediators in regulating B cell activity after allo-HCT.

The UPR consists of highly conserved signaling pathways that allow the cells to manage ER stress in response to the accumulation of unfolded or misfolded protein (12). There are three primary UPR mediators including IRE-1α, PERK, and ATF6 (13). Endoribonuclease activity of IRE-1α activates XBP-1 signaling by converting unspliced XBP-1 to spliced XBP-1 (XBP-1s) and also mediates regulated IRE1α-dependent decay (RIDD) of ER-associated mRNAs harboring structures and sequences similar to XBP-1 mRNA stem loops (14–16). It has been demonstrated that IRE-1α/XBP-1s signaling is required for normal B cell development and also pivotal for plasma cell differentiation, which can secrete large amounts of immunoglobulin (Ig) (17–21). While XBP-1s has been previously shown to promote immunoglobulin production and secretion by plasma cells, deletion of IRE-1α kinase or RNase activity, that results in impaired XBP-1s production but also blocked RIDD activity, does not significantly compromise the capability of plasma cells in producing and secreting immunoglobulin (22, 23). Since deletion of XBP-1s can enhance RIDD of immunoglobulin mRNAs through upregulating the expression levels and kinase/ribonuclease activity of IRE-1α leading to scarcity of immunoglobulin proteins, further targeting IRE-1α kinase/RNase activity by deleting IRE-1α or mutating serine 729 to alanine (S729A) in IRE-1α kinase activation loop indeed results in the recovery of immunoglobulin production and secretion by XBP-1s-deficient plasma cells (20, 22, 23). We identified a critical role of IRE-1α/XBP-1s signaling in B cells for the development of cGVHD through genetic and pharmacological inhibition of XBP-1s in mouse allo-HCT models (24). However, it is unclear whether RIDD plays an important role in reducing the severity of cGVHD.

Here, we identified the role of RIDD in B cell-mediated cGVHD pathogenicity. Deletion of XBP-1s reduced B cell activity and ability to stimulate allogeneic CD4 T cells in an RIDD-dependent manner which reduced the severity of cGVHD in the murine allo-HCT model. Our results showed that activating RIDD by targeting XBP-1s is a useful strategy to reduce cGVHD.

## Materials and Methods

### Mice

BALB/c (H-2^d^), and FVB (H-2^q^) mice were purchased from the National Cancer Institute (Frederick, MD). B cell conditional XBP-1^KO^ (XBP-1^flox/flox^CD19-Cre^+^), IRE-1α^KO^ (kinase domain (aa652-751) flanked by LoxP site (IRE-1α^flox/flox^CD19-Cre^+^), XBP-1/IRE-1α double KO (DKO) (XBP-1^flox/flox^IRE-1α^flox/flox^CD19-Cre^+^), and littermate wild-type control (XBP-1^flox/flox^CD19^-^) mice on a C57BL/6(B6, H-2^b^) background were generated as described before (20, 23, 25, 26). The S729A knock-in mouse model was generated as previously described (23). Mice were maintained at pathogen-free facilities in the American Association for Laboratory Animal Care–accredited Animal Resource Center at Medical University of South Carolina (MUSC) and the animal facility at the Houston Methodist Research Institute (HMRI). All animal experiments were approved by the Institutional Animal Care and Use Committees at MUSC and HMRI.

### Antibodies and Reagents

Antibodies were purchased as followed: Anti-CD4-V450, anti-CD8α-APCcy7, anti-B220-V450, anti-CD138-Pe-cy7, anti-CD86-Pe-cy5, anti-FAS-PE, anti-GL-7-APC, anti-CD40-APC, anti-MHCII-FITC, anti-IL-4-PE, anti-IL-5-PE, anti-IgM-Pe-cy7, anti-IgG1-APC, anti-IFN-r-Percp5.5, anti-TNF-α-PE, and anti-IL-17-Pe-cy7 were purchased from BD Biosciences (Franklin Lakes, NJ). Anti-PDL-1-biotin (eBioscience, San Diego, CA), anti-XBP-1s (Cell Signaling Technology, Danvers, MA), and anti-Rabbit IgG-FITC (Thermo Fisher Scientific, Waltham, MA) antibodies were purchased from commercial sources. Recombinant mouse IL-4 (PeproTech, Rocky Hill, NJ) and LPS (Sigma-Aldrich, St. Louis, MO) were purchased from the commercial companies. Goat F(ab’)2 Anti-Mouse IgM (1022-01, SouthernBiotech, Birmingham, AL) and anti-mouse CD40 (BE0016-2, BioXCell, Lebanon, NH) were commercially purchased from companies.

### Allogeneic Bone Marrow Transplantation

Recipient BALB/c mice (8-10 weeks old) were lethally irradiated at 650 – 700 cGy using X-RAD 320 irradiator (Precision X-ray Inc., North Brandford, CT). 5 × 10^6^ T cell depleted bone marrow (TCD-BM) cells were transplanted into recipient mice with or without 0.3 – 0.5 × 10^6^ splenocytes. Survival, body weight, and clinical scores of cGVHD from recipient mice were monitored as described previously (27). Clinical scores were calculated with the combination of 7 parameters established in the previous report (28) including weight loss, posture, activity, fur texture, skin integrity, diarrhea, and eye inflammation or conjunctivitis. Individual mice were scored 0 to 2 for each criterion and 0 to 12 overall. We arbitrarily considered mice falling under the score categories 0.5–3 as mild, 4–7 as moderate, and 8–12 as showing severe symptoms where scores ≥ 8 required euthanasia as a humane endpoint.

### *In Vitro* Mixed Lymphocyte Reaction

B cells purified from WT, XBP-1^KO^, IRE-1α^KO^, and DKO B6 mice were stimulated with LPS (1μg/ml) and IL-4 (10ng/ml) for 24 h. LPS and IL-4 were removed from culture plate wells. T cells from FVB mice were stained with Carboxyfluorescein diacetate succinimidyl ester (CFSE, Invitrogen, Molecular Probes, Inc., Eugene, OR) and co-cultured with pre-stimulated B cells for 3-4 days. T cell proliferation and cytokine expression were determined with flow cytometry analysis.

### Serum Immunoglobulin Detection

Using DNA from calf thymus (Sigma-Aldrich), we made double-strained DNA (dsDNA) (27). ELISA plates were coated with a 5μg/ml dsDNA overnight at 37°C. The plates were blocked with 1% BSA solution in PBS for 30 min. After blocking, serum or cell supernatant was added at a 1:10 to 1:100 ratio in PBS containing 0.05% Tween and 1% BSA. Plates were incubated at room temperature (RT) for 45 minutes and then washed. Biotin-conjugated IgM or IgG1 antibody (BD Bioscience) was added at a 1:4000 ratio and incubated for another 45 min in RT. Plates were then washed and added with streptavidin-HRP antibody (Invitrogen) at a 1:250 ratio and incubated for 45 min in RT. After washing the plates, TMB Substrate (eBioscience) was added to the plates. The reaction was stopped after 15 min using 1M phosphoric acid and the plates were read at 450nm.

### Statistics

Data were presented by means ± standard deviation (SD) or means ± standard error of the mean (SEM) and statistical analyses were performed by GraphPad Prism software, version 9. Statistics for GVHD scoring and mice weight were performed using two-way ANOVA with Tukey’s multiple comparison test. Comparison of the survival distributions of any given groups were done using log-rank test. One way analysis of variance (ANOVA) with the Tukey’s multiple comparison test was used for multiple groups comparisons unless otherwise stated. p < 0.05 is considered statistically significant.

## Results

### Inhibition of IRE-1α/XBP-1 Signaling Reduces B Cell Activation, Differentiation, and IgM Secretion Through RIDD-Dependent Manner

**Figure 1:**
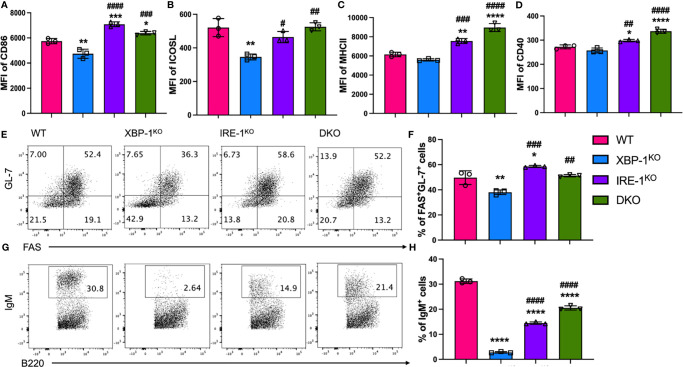
Roles of XBP-1s and IRE-1α in B cell activation, differentiation and IgM production *in vitro*. B cells were isolated from WT, XBP-1^KO^, IRE-1α^KO^, and IRE-1α^KO^/XBP-1^KO^ (double KO; DKO) mice and stimulated with 1 mu;g/ml LPS and 10 ng/ml IL-4 for 4 days. The cell surface expression levels of CD86 **(A)**, ICOSL **(B)**, MHCII **(C)**, and CD40 **(D)** were measured by flow cytometry analysis. Germinal Center B cells (FAS^+^GL-7^+^) were detected by flow cytometry **(E, F)**. B cells were stimulated with PMA and Ionomycin for another 4 hrs. B cells were intracellularly stained and analyzed for IgM production **(G, H)**. Data are representative graphs and flow cytometry plots of three independently repeated experiments. Data are shown as means ± SD. MFI, mean fluorescence intensity. Statistics were performed using ordinary one-way ANOVA with Tukey’s multiple comparison test. *p < 0.05, **p < 0.005, ***p < 0.0005, and ****p < 0.0001 when compared to WT. ^#^p < 0.05, ^##^p < 0.005, ^###^p < 0.0005, and ^####^p < 0.0001 when compared to XBP-1^KO^ group.

We hypothesized that enhanced RIDD resulting from XBP-1s deletion is an important factor to reduce B cell pathogenicity in cGVHD development. To test this hypothesis, we compared the activation, differentiation, and immunoglobulin (Ig) production of B cells from WT or genetically modified mice that have XBP-1s, IRE-1α, or XBP-1s/IRE-1α deficiency specifically on their B cells. Since IRE-1α/XBP-1 signaling is known to be activated by LPS and IL-4 stimulation, which are also commonly presented in BMT recipients (29), we stimulated B cells with them. First, we measured the expression of XBP-1s, and confirmed that it was eliminated in XBP-1^KO^, IRE-1α^KO^, and DKO B cells compared to WT B cells (**Supplementary Figure 1**) (23). Activated B cells increase the expression of MHCII and costimulatory molecules including CD86, CD40, and ICOS. The expression of CD86 and ICOSL was reduced on XBP-1^KO^ B cells compared with WT counterparts, but it was increased on IRE-1α^KO^ and DKO B cells where RIDD was also impeded (**Figures 1A, B**). The expression levels of MHCII and CD40 were significantly increased in IRE-1α^KO^ and DKO B cells compared to WT B cells (**Figures 1C, D**). Germinal center (GC) B cell development was also significantly lower in XBP-1^KO^ B cells, but it was reversed in IRE-1α^KO^ and DKO groups (**Figures 1E, F**). IgM is the first antibody produced by plasma cells and its mRNA is the representative substrate of RIDD in B cells (22). B cells from XBP-1^KO^ mice expressed a significantly lower level of IgM compared to WT B cells, which is related to the reduced GC B cell population by XBP-1s deletion (**Figures 1E, F**) and also confirms that RIDD was increased in XBP-1s-deficient B cells (**Figures 1G, H**). Therefore, ablation of RIDD by deleting IRE-1α in IRE-1α^KO^ or DKO B cells restored the IgM expression compared to XBP-1^KO^ B cells (**Figures 1G, H**). On the other hand, intracellular IgG1 expression was not dramatically affected in XBP-1^KO^, IRE-1α^KO^ or DKO B cells (**Supplementary Figures 2A, B**). XBP-1^KO^ B cells showed reduced IL-4/IL-5 expression but such a reduction was not restored by IRE-1α deletion suggesting these cytokines are not regulated by RIDD (**Supplementary Figures 2C, D**).

XBP-1s is also required for the proper signaling through the B cell receptor (BCR) (20). BCR is composed of a membrane-bound immunoglobulin (IgM or IgD) and the disulfide-linked Igα/Igβ heterodimer. To induce BCR signaling, we stimulated B cells with anti-IgM and anti-CD40 for stable activation. Similar to stimulation with LPS and IL-4, XBP-1s deficiency repressed the B cell activation, GC cell differentiation and sIgM expression but these phenotypes were reversed by RIDD inhibition in IRE-1^KO^ and DKO B cells after BCR stimulation (**Supplementary Figure 3**). Taken together, these data indicate that inhibition of XBP-1s in B cells reduces B cell activation and differentiation through both RIDD-dependent and -independent manners.

### RIDD Is Essential for the Ability of XBP-1s-Deficient B Cells to Activate T Cells

**Figure 2:**
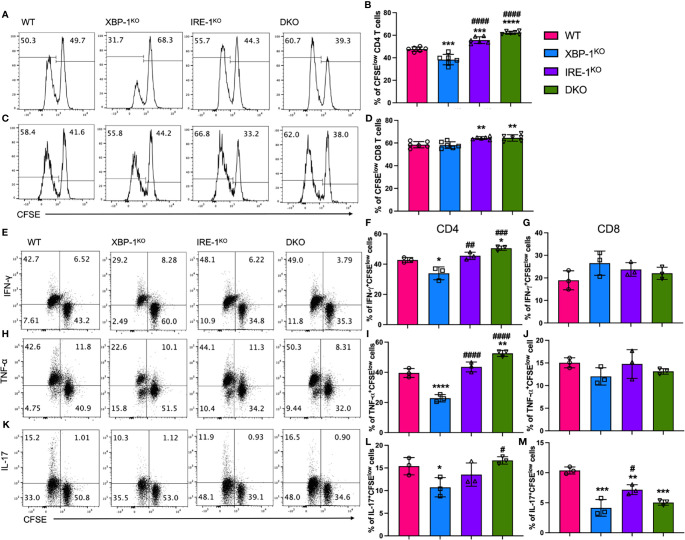
Effects of XBP-1s and IRE-1α on B cell ability to stimulate allogeneic T cells *in vitro*. B cells isolated from WT, XBP-1^KO^, IRE-1α^KO^, and DKO on B6 background mice are stimulated with 1 μg/ml LPS and 10 ng/ml IL-4 for 24 hrs. T cells were isolated from FVB mice and labeled with CFSE and then incubated with activated B cells for another 4 days. The representative flow panels of CFSE dilution and percentages of CFSE diluted cells on CD4 **(A, B)** and CD8 **(C, D)** T cells. T cells were stimulated with PMA and Ionomycin for 4 h before intracellular cytokine staining for cytokine detection. The representative flow panels and the percentages of IFN-γ **(E–G)**, TNF-α **(H–J)** or IL-17 **(K–M)** among proliferated (CFSE^low^) CD4 and CD8 T cells were shown. Data show representative flow cytometry plots and graphs from three independently repeated experiments. Statistics were performed using ordinary one-way ANOVA with Tukey’s multiple comparison test. *p < 0.05, **p < 0.005, ***p < 0.0005, and ****p < 0.0001 when compared to WT. ^#^p < 0.05, ^##^p < 0.005, ^###^p < 0.0005, and ^####^p < 0.0001 when compared to XBP-1^KO^ group.

Activated B cells are important APCs in alloantigen-rich immunologic microenvironment, which can prime allogenic T cells in ongoing cGVHD (30). Therefore, we tested whether and how RIDD may affect B cell activity to stimulate allogeneic T cells *in vitro*. WT T cells from FVB mice were stimulated with allogeneic B cells from different B6 mice (WT, XBP-1^KO^, IRE-1α^KO^, or DKO). T cells, particularly CD4 T cells, had a reduced proliferation reflected by a decreased CFSE dilution when stimulated with XBP-1^KO^ B cells, but such a reduction was not observed in those stimulated with IRE-1α^KO^ or DKO B cells (**Figures 2A, B**). The proliferation of CD8 T cells was slightly increased by IRE-1α^KO^ or DKO B cell stimulation (**Figures 2C, D**). Moreover, CD4 T cells, but not CD8 T cells, expressed significantly less inflammatory cytokines including IFN-γ (**Figures 2E–G**) and TNF-α (**Figures 2H–J**) when stimulated by XBP-1^KO^ B cells. Both CD4 and CD8 T cells produced decreased levels of IL-17A when stimulated with XBP-1s-deficient B cells (**Figures 2K–M**). However, similar or even higher levels of cytokines were observed when CD4 T cells were stimulated with IRE-1α^KO^ and DKO B cells in which not only XBP-1s was not expressed but also RIDD was ablated. Collectively, our results indicate that activated RIDD in XBP-1^KO^ B cells is essential for optimal activation of allogeneic CD4 and potentially CD8 T cells.

### XBP-1s Deficiency on B Cells Decreases Severity of cGVHD Through RIDD

**Figure 3:**
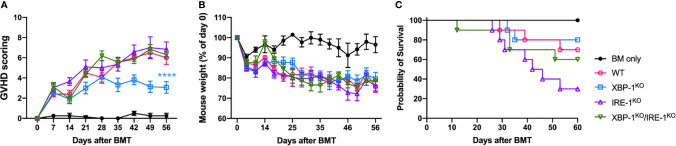
Long-term impact of XBP-1s and IRE-1α on B cell pathogenicity in the induction of cGVHD. BALB/c mice were lethally irradiated and transplanted with 5 x 10^6^ T cell-depleted bone marrow (TCD-BM) cells from WT (n = 4) or TCD-BM from WT, XBP-1^KO^, IRE-1α^KO^, and DKO mice on a B6 background with 0.35 - 0.5 x 10^6^ splenocytes (n = 40). Recipient mice were monitored for the GVHD scoring **(A)**, weight loss **(B)**, and mortality **(C)** until 60 days after BMT. Data show results from two out of three independently repeated experiments. 22 recipient mice were used for each experiment. Statistics for scoring and weight were performed using two-way ANOVA with Tukey’s multiple comparison test. Comparisons of the survival distributions of any given groups were done using log-rank test. ****p < 0.0001 when compared to WT.

We showed that B cell activation and differentiation can be regulated by RIDD activation resulting in altered alloreactive T cell activation. Here, we investigated the role of XBP-1s deficiency-mediated activation of RIDD in the prevention of cGVHD using an MHC-mismatched murine BMT (B6 to BALB/c) model. The recipients transplanted with XBP-1s-deficient donor-graft showed significantly reduced cGVHD severity (**Figures 3A–C**). Induction of follicular helper T cell (T_FH_) is required for cGVHD development by supporting GC formation and maintenance (31). We found that T_FH_ phenotype was notably increased in the recipients of IRE-1α^KO^ or DKO graft (**Supplementary Figures 4A, B**). Besides, donor CD4 T cells in the recipients of XBP-1^KO^ graft produced a significantly lower level of IFN-γ and IL-17, but T cells from those of RIDD-ablated IRE-1α^KO^ or DKO grafts expressed similar, or even higher levels of cytokines compared to WT (**Supplementary Figures 4C–F**). These data support that XBP-1s deficiency-mediated activation of RIDD in B cells affects the T cell pathogenicity and consequently regulates the cGVHD severity in the long-term period.

### RIDD Regulates B Cell Activation and cGVHD Pathogenesis

**Figure 4:**
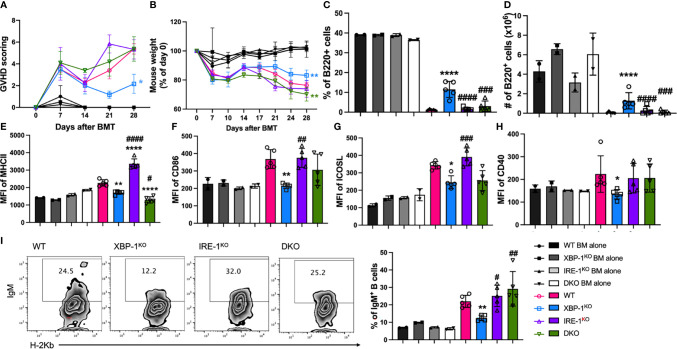
Effect of XBP-1s and IRE-1α on B cell activation and cGVHD pathogenicity. BALB/c mice were lethally irradiated and transplanted with 5 x 10^6^ TCD-BM cells from WT, XBP-1^KO^, IRE-1α^KO^, and DKO mice on a B6 background with (n = 20) or without (n = 8) 0.35 - 0.5 x 10^6^ splenocytes. GVHD scoring **(A)** and mouse weight **(B)** were monitored during the experiment. Subsets of recipient mice were euthanized on day 28, and spleens were dissected and processed into single-cell suspension. The percentages of B220^+^ B cells **(C)** and the expression levels of MHCII **(D)**, CD86 **(E)**, ICOSL **(F)**, and CD40 **(G)** were detected by flow cytometry analysis. Intracellular expression of IgM **(H, I)** was also determined using flow cytometry. Data show the representative result from three independently repeated experiments. 28 recipient mice were used for each experiment. Statistics were performed using two-way ANOVA with Tukey’s multiple comparison test for **(A, B)** One-way ANOVA with Tukey’s multiple comparison test was used for others. *p < 0.05, **p < 0.005, and ****p < 0.0001 when compared to WT. ^#^p < 0.05, ^##^p < 0.005, ^###^p < 0.0005, and ^####^p < 0.0001 when compared to XBP-1^KO^ group.

To determine the cellular mechanisms associated with cGVHD development, we used B6 to BALB/c cGVHD model and euthanized recipient mice at 4 weeks after allo-BMT for analyses of donor B and T cell responses. Recipients transplanted with allogeneic grafts from B cell-specific XBP-1^KO^ donors showed attenuated severity of GVHD represented by reduced GVHD scoring and improved weight loss (**Figures 4A, B**). However, the recipients with IRE-1α^KO^ or DKO donor grafts showed similar levels of GVHD development compared to WT counterparts (**Figures 4A, B**). The recipients of XBP-1^KO^ donor-grafts had higher frequencies of donor-derived B cells in their spleens, but this was not the case in the recipients transplanted with IRE-1α^KO^ or DKO donor-grafts where RIDD was also ablated in donor B cells (**Figures 4C, D**). B cells from the recipients with XBP-1^KO^ donor-grafts showed significantly decreased expression levels of MHCII, CD86, ICOSL, and CD40, which were restored in B cells from those with IRE-1α^KO^ or DKO donor-grafts (**Figures 4E–H**). In the recipients with XBP-1^KO^ donor-grafts, B cell showed significantly decreased expression of IgM (**Figure 4I**). Ablation of RIDD in B cells in IRE-1α^KO^ or DKO donor-grafts enhanced IgM production compared to those in XBP-1^KO^ donor-grafts (**Figure 4I**). Consistent with *in vitro* data, IgG1, IL-4 and IL-5 expression levels were reduced in XBP-1s-deficient B cells, but such reductions could not be restored by further deleting IRE-1α (**Supplementary Figure 5**). These data indicate that RIDD is a key mediator, which can reduce cGVHD in the context of targeting XBP-1s.

### RIDD Regulates the Capabilities of B Cells in Activating T Cells During cGVHD

**Figure 5:**
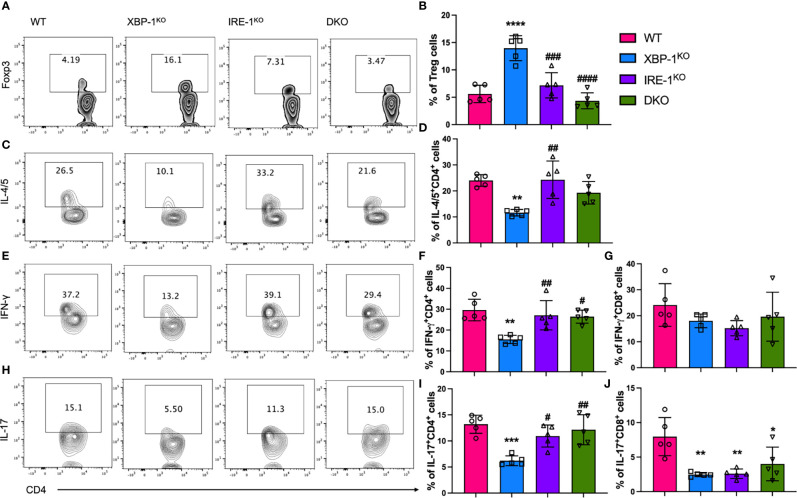
Effect of XBP-1s and IRE-1α on B cell ability to stimulate allogeneic T cells during cGVHD. Lethally irradiated BALB/c mice were transplanted with 5 x 10^6^ TCD-BM cells from WT, XBP-1^KO^, IRE-1α^KO^, and DKO mice on a B6 background with 0.35 - 0.5 x 10^6^ splenocytes (n=20). Recipient mice were euthanized on day 28 after BMT and single cells were isolated from recipient spleens. The expression of Foxp3 transcription factor **(A, B)** was measured by flow cytometry analysis. The cytokine levels in CD4 and CD8 T cells including IL-4/IL-5 **(C, D)**, IFN-γ **(E–G)**, and IL-17 **(H–J)** were determined after intracellular staining by flow cytometry analysis. Data show the representative result from three independently repeated experiments. 20 recipient mice were used for each experiment. Statistics were performed using two-way ANOVA with Tukey’s multiple comparison test. *p < 0.05, **p < 0.005, ***p < 0.0005, and ****p < 0.0001 when compared to WT. ^#^p < 0.05, ^##^p < 0.005, ^###^p < 0.0005, and ^####^p < 0.0001 when compared to XBP-1^KO^ group.

We observed that MHCII expression and costimulatory activity of B cells were regulated by RIDD, which can affect T cell pathogenicity in the induction of cGVHD. Therefore, we tested donor T cell activation in the recipients transplanted with different donor grafts. We found that Treg differentiation was increased in T cells in the recipients of XBP-1^KO^ donor-grafts (**Figures 5A, B**). Furthermore, Treg frequency was reversed in T cell populations from the recipients transplanted with IRE-1α^KO^ or DKO donor-grafts, consistent with CD86 expression on corresponding types of donor B cells (**Figure 4F**). Opposite to Tregs with a suppressive role, T helper (Th)1, Th2, and Th17 cells play a pathogenic role in GVHD development (32, 33). We measured the presence of Th2 (IL-4/IL-5+), Th1 (IFN-γ+), and Th17 (IL-17+) T helpers in donor T cell populations from the recipients transferred with various donor-grafts. In XBP-1^KO^ donor-graft transplanted mice, T cells showed decreased Th1, Th2, and Th17 differentiation, which was reversed in the recipients transplanted with IRE-1α^KO^ or DKO donor grafts (**Figures 5C–F, H, I**). On the other hand, there was no significant difference in IFN-γ secreting type 1 CD8 T cell (Tc1) differentiation among the four groups (**Figure 5G**). T cells from the recipients transplanted with XBP-1^KO^ or IRE-1α^KO^ donor grafts were significantly less capable of differentiating into IL-17-producing CD8 T cells (Tc17) (**Figure 5J**). Collectively, these results suggest that activated RIDD as a result of XBP-1s deficiency specifically in B cells can change CD4 T cell differentiation during cGVHD.

### RIDD Affects Activation and Differentiation of B Cells, and Their Ability to Activate T Cells

**Figure 6:**
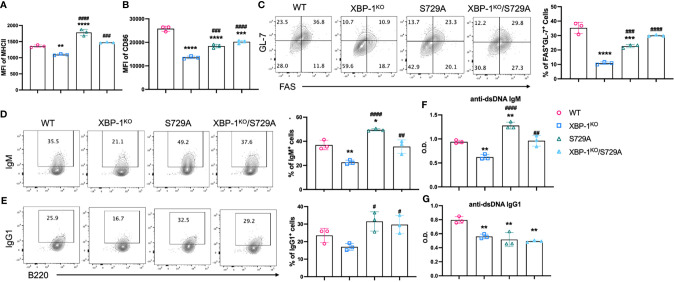
Roles of XBP-1s and the S729A mutation of IRE-1α in B cell activation, differentiation, and IgM production *in vitro*. B cells were isolated from WT, XBP-1^KO^, S729A, and XBP-1^KO^/S729A mice and stimulated with anti-IgM F(ab’)2 (10 mu;g/ml) and anti-CD40 (10 mu;g/ml) for 48 hrs. The expression of MHCII **(A)** and CD86 **(B)** were detected by flow cytometry. The percentages of germinal center B cells (FAS^+^GL-7^+^) were presented **(C)**. **(D, E)** B cells were stimulated with PMA and Ionomycin for 4 hrs on day 4. Intracellular levels of IgM **(D)** and IgG1 **(E)** were detected by flow cytometry analysis. Cell culture media were also collected, and anti-dsDNA autoantibodies were detected using ELISA **(F, G)**. Statistics were performed using two-way ANOVA with Tukey’s multiple comparison test. *p < 0.05, **p < 0.005, ***p < 0.0005, and ****p < 0.0001 when compared to WT. ^#^p < 0.05, ^##^p <0.005, ^###^p < 0.0005, and ^####^p < 0.0001 when compared to XBP-1^KO^ group.

The kinase domain of IRE-1α is decisive for the ribonuclease activity of IRE-1α, and especially phosphorylation of the S729 residue is important for RIDD, because IRE-1α carrying the S729A mutation shows ablated RIDD activity but unabated activity in splicing XBP-1 mRNA (23). First, we confirmed that XBP-1s signaling is not affected by S729A mutation in B cells (**Supplementary Figure 6**). To evaluate the role of RIDD in B cells, we investigated activation and differentiation of B cells from S729A and XBP-1^KO^/S729A mice and compared them with B cells from WT and XBP-1^KO^ mice, after B cells were treated with F(ab’)2 and anti-CD40. As we expected, the reduced expression levels of MHCII and CD86 resulted from XBP-1s deficiency were reversed in XBP-1^KO^/S729A B cells (**Figures 6A, B**). The formation of GC B cells was also decreased when XBP-1s is deleted in B cells, but it was restored by further introducing S729A into IRE-1α in XBP-1^KO^ B cells (**Figure 6C**). Decreased IgM-positive B cells and reduced IgM levels secreted into culture media resulted from XBP-1s deficiency were also reversed by further introducing the S729A mutation into IRE-1α (**Figures 6D, F**). However, reduced IgG1 expression levels resulted from XBP-1s deficiency were not reversed by the S729A mutation of IRE-1α (**Figures 6E, G**), similar to our IRE-1α kinase deletion results (**Supplementary Figures 5A, B**). B cells with S729A mutation showed even higher MHCII expression and IgM secretion compared to WT B cells after stimulation with F(ab’)2 and anti-CD40 (**Figures 6A, F**). However, B cells from S729A mice have already shown curtailed activity and reduced GC B cell formation compared to WT B cells when we stimulate B cells with LPS and IL-4 (**Supplementary Figures 7A–E**) while IgM expression is still increased in S729A B cells (**Supplementary Figure 7F**). These data showed that S729A B cells may respond differently to TLR4 signaling compared to BCR signaling. Taken together, these results suggest that RIDD is essential for reduced activation and differentiation of XBP-1s-deficient B cells.

We next tested whether RIDD inhibition by introducing the S729A mutation to IRE-1α regulates B cell ability to stimulate allogeneic T cells. CFSE-labeled allogeneic T cells were stimulated with B cells from WT, XBP-1^KO^, S729A, or XBP-1^KO^/S729A B6 mice *in vitro*. Similar to the IRE-1α^KO^ and DKO (**Figures 2A, B**), B cells from XBP-1^KO^ mice carrying the S729A mutation did not reduce CD4 T cell proliferation (**Supplementary Figures 8A, B**). On the other hand, there was no significant difference in CD8 T cell proliferation among all four groups (**Supplementary Figures 8C, D**). CD4 T cells stimulated with XBP-1s-deficient B cells produced significantly less IFN-γ, IL-4 and IL-5, but the levels of these cytokines were restored by further introduction of the S729A mutation of IRE-1α into XBP-1^KO^ B cells (**Supplementary Figures 8E, F, H, I**). S729A knock-in B cells showed reduced expression of MHCII and costimulatory molecules when stimulated with LPS and IL-4 (**Supplementary Figures 7A–D**). Consequently, coculture of T cells with LPS and IL-4 stimulated allogeneic S729A B cells exhibited reduced proliferation and cytokine production in CD4 but not CD8 T cell populations (**Supplementary Figures 8 E–J**). Taken together, these data support inhibition of XBP-1s in B cells can reduce the allogeneic T cell activity through activating RIDD.

### RIDD Affects the Severity of cGVHD

**Figure 7:**
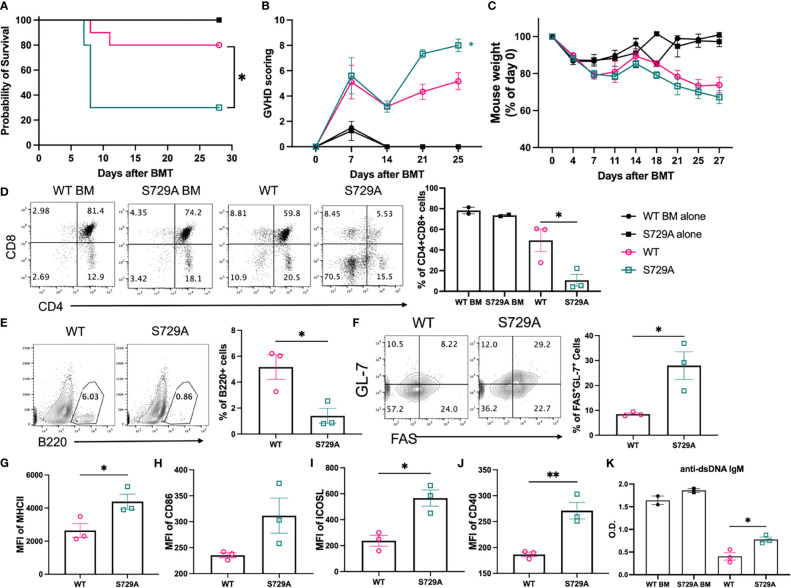
Effect of S729A mutation on B cell activation and cGVHD pathogenicity. BALB/c mice were lethally irradiated and transplanted with 5 x 10^6^ TCD-BM cells from WT and S729A mice on a B6 background with (n = 10) or without (n = 4) 0.12 x 10^6^ B cells from WT or S729A mice and 0.18 x 10^6^ B cell deleted WT splenocyte. Survival **(A)**, GVHD score **(B)** and mouse weight **(C)** were monitored during the experiment. Subsets of recipient mice were euthanized on day 28, and thymi and spleens were dissected and processed into single-cell suspension. CD4 and CD8 double positive cell percentages in thymus were determined using flow cytometry **(D)**. The percentages of B220^+^ B cells **(E)**, germinal center B cells (FAS^+^GL-7^+^) **(F)**, the expression of MHCII **(G)**, CD86 **(H)**, ICOSL **(I)**, and CD40 **(J)** were detected by flow cytometry analysis. Serum isolated from recipients was collected on day 28 and assayed for anti-dsDNA autoantibodies using ELISA **(K)**. **(A–C)** shows combined data from two independently repeated experiments. **(D–K)** shows representative data from two independently repeated experiments. 14 recipient mice were used for each experiment. Comparison of the survival distributions in **(A)** was done using log-rank test. Statistics were performed using two-way ANOVA with Tukey’s multiple comparison test for **(B, C)** One-way ANOVA with Tukey’s multiple comparison test was used for comparing multiple groups and 2-tailed Student *t* test was used for comparing between two groups. *p < 0.05, **p < 0.005, and ****p < 0.0001 when compared to WT. ^#^p < 0.05, ^##^p < 0.005, ^###^p < 0.0005, and ^####^p < 0.0001 when compared to XBP-1^KO^ group.

**Figure 8:**
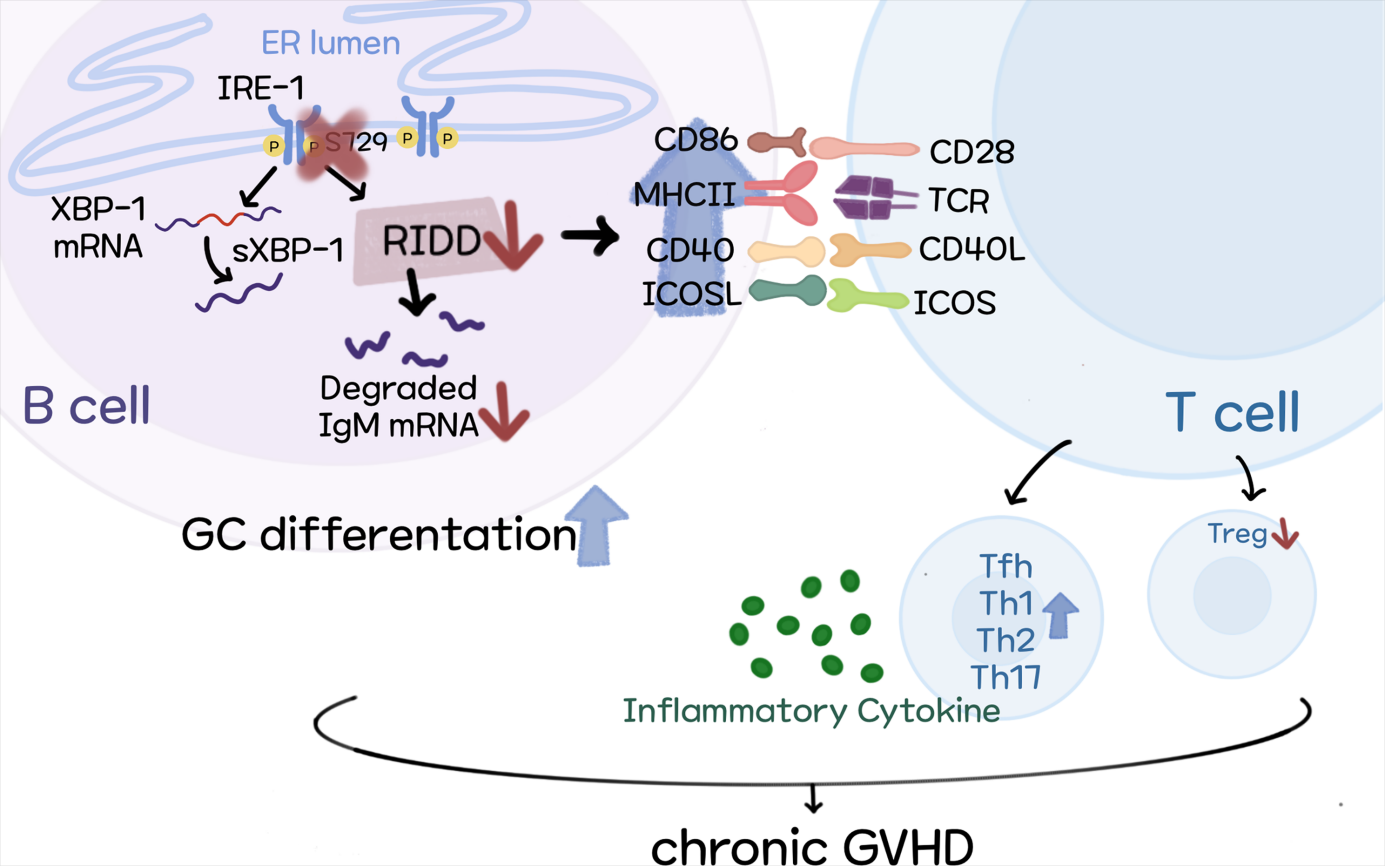
Schematic diagram of our proposed model.

We next wanted to directly test the role of RIDD activity in B cell pathogenicity in the induction of cGVHD by taking advantage of the S729A mutation that specifically inhibits RIDD activity of IRE-1α while preserving its ability in splicing XBP-1 (**Supplementary Figure 6**). Because S729A is germline mutation, we isolated B cells from IRE-1α S729A mutant or control mice and compared their ability to induce cGVHD. The recipients transferred with S729A mutant B cells developed more severe cGVHD as reflected by decreased survival, increased GVHD scores, and weight loss (**Figures 7A–C**). The recipients of S729A mutant B cells demonstrated significantly increased thymus damage (**Figure 7D**) and reduced frequencies of the donor-derived B cells in the spleens (**Figure 7E**). These recipients also displayed a significantly increased percentage of GC B cells, exhibited higher expression levels of MHCII, CD86, ICOSL, and CD40 (**Figures 7F–J**), and produced significantly higher levels of anti-dsDNA IgM (**Figure 7K**) as compared with WT B cells. Taken together, these data indicate that RIDD activity of B cells contributes to a diverse repertoire of B cell function including activation, differentiation, and immunoglobulin production that resulted in reduced cGVHD (**Figure 8**).

## Discussion

We previously demonstrated that targeting XBP-1s in B cells efficiently prevented cGVHD by reducing activation and differentiation of B cells (24). However, how XBP-1s deficiency regulates B cell response in cGVHD was not fully defined. Since the absence of XBP-1s in B cells has been reported to confer the upregulation of IRE-1α expression accompanied by increased kinase and ribonuclease activity, we hypothesized that RIDD can be a key mediator in response to targeting XBP-1s for cGVHD prevention. IRE-1α has kinase and RNase domains in the cytoplasmic region, and under ER stress, autophosphorylation of the kinase domain results in RNase activity (34). In this study, we used B cell specific IRE-1α kinase domain deleted mice to determine how RIDD impacts the role of XBP-1s deficiency in B cells and found that deletion of IRE-1α kinase activity attenuated the effect of targeting XBP-1s in the prevention of cGVHD. *In vitro* assay revealed that the expression of MHCII and costimulatory molecules are regulated by RIDD in B cells and consequently alloreactive stimulation of T cells is attenuated by activated RIDD in XBP-1s-deficient B cells.

We confirmed that RIDD was significantly increased in XBP-1s-deficient B cells through IgM expression (**Figure 1**). However, the absence of IRE-1α did not fully restore IgM production to the levels of WT B cells (**Figure 1**). We reason that IRE-1α-XBP-1s signaling is also needed to promote plasma cell differentiation in an RIDD-independent manner, thus insufficient differentiation lowers the IgM expression in IRE-1α KO or DKO B cells compared to WT counterparts. Increased splenic germinal centers that significantly enhance Tfh and GC B cell differentiation have been reported to be closely correlated with cGVHD development (31, 35). Moreover, inducible ICOS/ICOSL and CD40L/CD40 signaling between Tfh cells and B cells are essential to initiate a germinal center reaction and promote cGVHD (36, 37). We found that the expression of ICOSL and CD40 were repressed by XBP-1s deletion but restored in IRE-1α^KO^ or DKO B cells (**Figure 1**), suggesting that RIDD activation can suppress ICOSL and CD40 expression on B cells likely through an indirect mechanism. Accordingly, GC B cell and Tfh differentiation were also modulated by RIDD activation (**Figure 1** and **Supplementary Figure 4**).

Donor B cell-derived antibodies have been directly implicated in cGVHD progress by augmenting the fibrosis of target organs and inflammatory T cell infiltration (6, 38). RIDD has been shown to regulate IgM and IgG2b expression while IgG1 response relied on a different downstream element of IRE-1α/XBP-1s signaling (22). Besides, S729A mice with repressed RIDD activity have also been reported to produce increased serum IgM and IgG2b levels in response to immunization (23). Likewise, *in vitro* assay revealed that IgM expression was partially restored in IRE-1α^KO^ and DKO B cells (**Figures 1G–H**). In murine GVHD model, IgM production was more obviously restored in IRE-1α^KO^ and DKO B cells in transplanted recipients (**Figure 4I**) while IgG1 was curtailed to a level similar to the XBP-1^KO^ group (**Supplementary Figures 5A, B**). These data implicate that RIDD plays a critical role for plasma cells to reduce the production of IgM and certain subclasses of IgG in GVHD development. It has been suggested that deposition of IgG can lead to infiltration and activation of T cells and macrophages resulting in the cGVHD progress (6, 38). The role of secretory IgM on cGVHD has not been defined yet, however, it was reported that both IgG and IgM were frequently found in the basal epidermis in both acute and chronic GVHD patient biopsies, and their number was positively related to the degree of epidermal necrosis observed in histologic section (39). It has also been reported that patients with hyper IgM syndrome are prone to exposure the autoimmune disease (40, 41). Although one report suggests that inhibition of secretory IgM can induce more autoreactive IgG resulting in the more severe autoimmune disease in lupus-prone lymphoproliferative mice (42), it is possible that that sIgM may play a different role in cGVHD. In addition, the previous paper suggested sIgM suppressed the development of IgG, but we consider that increased IgG development may be because of the increased Ig class switching by restricting IgM secretion. In our mouse GVHD model, the recipients transferred with XBP-1s deficient B cells showed reduced sIgM expression, but they didn’t show increased IgG1 expression (**Figure 4I** and **Supplementary Figure 5**). Since IgM is the main target of RIDD in B cells and their expression in serum positively correlated with the severity of the disease, we reason deposition of IgM may play an essential role in the development of cGVHD. Further studies are needed to determine this hypothesis.

Alloreactive T cells need to be primed by APCs to initiate GVHD, and specifically, CD86 and CD40 mediated-costimulation from APCs has been demonstrated to play an essential role in eliciting cGHVD (43, 44). In light of our *in vivo* and *in vitro* data on costimulatory molecules, RIDD inhibition by deleting IRE-1α restored the expression of CD86 and CD40 in XBP-1s-deficient B cells (**Figure 1** and **4**). Besides, the expression of MHCII on B cells that present antigens to activate CD4 T cells was also modulated by RIDD (**Figures 1A**, **4E**). As a result, we demonstrated that alloreactivity of T cells, especially CD4 T cells, can be recovered by suppressing RIDD in XBP-1s-deficient B cells (**Figure 2**). It has been assumed that Th1 cells play a dominant role in acute GVHD, whereas Th2 cells are important for cGVHD. However, recent studies have reported that cytokines from Th1 (IFN-γ) and Th17 (IL-17) cells also play important roles in cGVHD progression (45, 46). Similar to previous reports, our data demonstrated that the differentiation of Th1, Th2, and Th17 was reduced upon interaction with XBP-1s-deficient B cells but restored by suppressing RIDD *via* further deleting IRE-1α, which in turn contributed to the severity of cGVHD (**Figure 5**). Besides, poor Treg reconstitution after allo-HCT has been suggested to result in the expansion of Th1 and Th17 cells that released proinflammatory cytokines and increased the risk of cGVHD (47, 48). We determined that CD28 mediated-Lck signaling suppresses the generation of iTreg (49). Based on these reports, we reason altered CD86 expression by RIDD increased CD28 costimulatory signaling in T cells, affected Treg differentiation during cGVHD development, and consequently influenced Th1 and Th17 differentiation (**Figure 5A**).

It has been reported that phosphorylation of the S729 residue in IRE-1α contributes to the upregulation of RIDD in XBP-1s-deficient B cells (23). We demonstrate that RIDD triggered by XBP-1s deletion could also be diminished by introducing the S729A mutation (**Figure 6D** and **Supplementary Figure 7F**). As a result, activation, differentiation, and alloreactivity of B cells were also restored in S729A/XBP-1^KO^ B cells (**Figure 6** and **Supplementary Figure 8**). RIDD inhibition with intact XBP-1s expression in S729A B cells resulted in much higher IgM and MHCII expression compared to WT, XBP-1^KO^ and S729A/XBP-1^KO^ B cells (**Figure 6** and **Supplementary Figure 7**), highlighting the role of RIDD in suppressing GVHD. Unexpectedly, S729A B cells showed reduced costimulatory factor expression and GC formation (**Figures 6B,C** and **Supplementary Figures 7A–E**). Since RIDD is important in maintaining basal ER homeostasis in B cells, we surmise that the increased accumulation of RIDD target molecules including IgM µ chain mRNA may compete for ribosomes and limit the expression of costimulatory factors in S729A B cells. Besides, since S729A B cells still produced XBP-1s, we interpret that these B cells may upregulate some chaperon genes under RIDD inhibition. Although S729 is located in the activation loop of IRE-1α, phosphorylation of IRE-1α is not essential for splicing the XBP-1 mRNA (23). Consistently, we also found that mutating the S729 phosphorylation site of IRE-1α did not limit B cells to produce XBP-1s. Altogether, we provide direct evidence showing that selective inhibition of RIDD activity of IRE-1α increases B cell pathogenicity in cGVHD induction (**Figure 7**).

To sum up, we demonstrate the mechanisms by which targeting XBP-1s alleviates cGVHD development. Our results indicate that activated RIDD resulted from XBP-1s deficiency may be responsible for reduced pathogenicity of B cells in the development of cGVHD, possibly through reducing IgM secretion and limiting B cell activation and differentiation (**Figure 8**).

## Data Availability Statement

The original contributions presented in the study are included in the article/**Supplementary Material**. Further inquiries can be directed to the corresponding authors.

## Ethics Statement

The animal study was reviewed and approved by The Institutional Animal Care & Use Committee.

## Author Contributions

Contribution: H-JC participated in research design, execution of experiments, statistical analysis and interpretation of data, and manuscript writing. C-HT generated mouse models, prepared mouse spleens and bone marrow for transplantation, interpreted data, and manuscript editing. LT, YW, MS, TT, and SS participated in conducting experiment and acquiring data. C-CH and X-ZY designed research, interpreted data, and edited the manuscript. All authors contributed to the article and approved the submitted version.

## Funding

This work was supported in part by grants from the National Institutes of Health, National Institute of Allergy and Infectious Diseases (R01 Al118305 to X-ZY), National Heart, Lung, and Blood Institute (R01 HL140953 to X-ZY), and National Cancer Institute (R01 CA163910 to C-CH). This publication was also supported in part by South Carolina Research Center of Economic Excellence and the Cell Evaluation & Therapy Shared Resource, Hollings Cancer Center, and Medical University of South Carolina (P30 CA138313).

## Conflict of Interest

The authors declare that the research was conducted in the absence of any commercial or financial relationships that could be construed as a potential conflict of interest.

## Publisher’s Note

All claims expressed in this article are solely those of the authors and do not necessarily represent those of their affiliated organizations, or those of the publisher, the editors and the reviewers. Any product that may be evaluated in this article, or claim that may be made by its manufacturer, is not guaranteed or endorsed by the publisher.
