# Supplementary material for: XBP-1s Promotes B Cell Pathogenicity in Chronic GVHD by Restraining the Activity of Regulated IRE-1α-Dependent Decay
Source: Front Immunol. 2021 Oct 1;12:705484. doi: 10.3389/fimmu.2021.705484 (PMC8517405; doi:10.3389/fimmu.2021.705484)
Supplement: Supplementary file 1 [file DataSheet_1.docx]

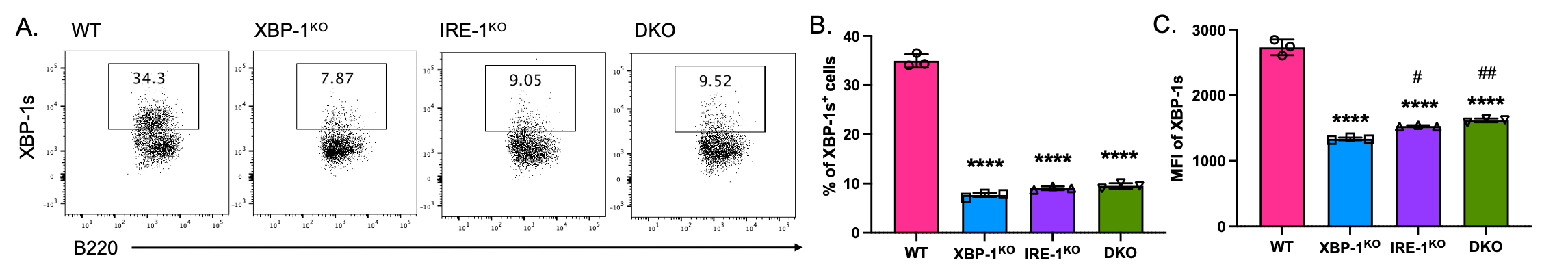


**Supplementary Figure 1. XBP-1s expression in XBP-1^KO^, IRE-1α^KO^, and DKO B cells.** WT, XBP-1^KO^, IRE-1α^KO^, and IRE-1α^KO^/XBP-1^KO^ (double KO; DKO) naïve B cells were stimulated with 1 𝜇g/ml LPS and 10 ng/ml IL-4 for 4 days and analyzed for the expression of spliced XBP-1 by flow cytometry. (A and B) Percentages of XBP-1s-expressing B cells. (C) Mean fluorescence intensity (MFI) of XBP-1s in B cells. Ordinary one-way ANOVA using Tukey’s multiple comparison test was used for panel B. **** p < 0.0001 when compared to WT. #p <0.05 and ## p < 0.005 when compared to XBP-1KO group.


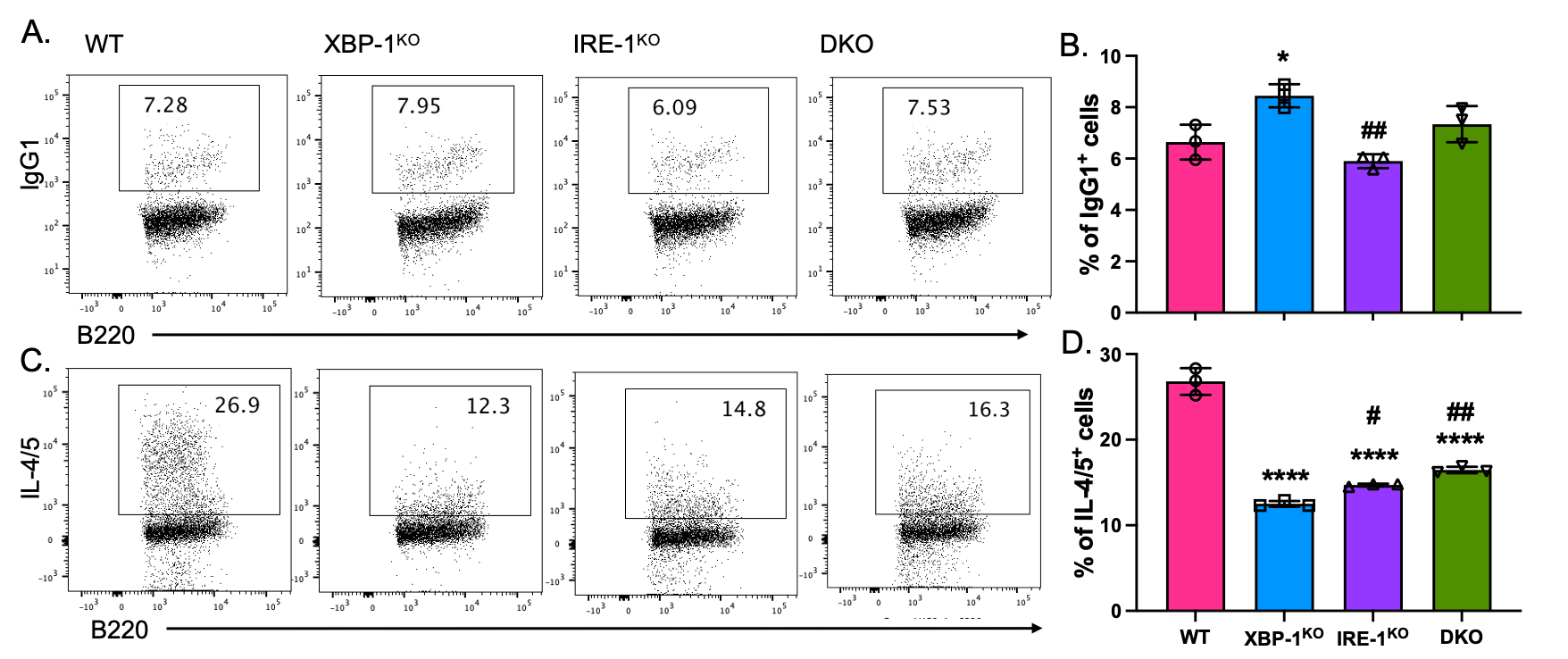


**Supplementary Figure 2. Roles of XBP-1s and IRE-1α in IgG1 and IL-4/5 production by B cells in vitro.** WT, XBP-1^KO^, IRE-1α^KO^, and IRE-1α^KO^/XBP-1^KO^ (double KO; DKO) naïve B cells were stimulated with 1 𝜇g/ml LPS and 10 ng/ml IL-4 for 4 days. B cells were stimulated for another 4 hrs with PMA and Ionomycin. Intracellular levels of IgG1 (A and B), IL-4/IL-5 (C and D) were determined by flow cytometry. Statistics were performed using ordinary one-way ANOVA with Tukey’s multiple comparison test. **** p < 0.0001 when compared to WT. # p < 0.05 and ## p < 0.005 when compared to XBP-1^KO^ group.

*
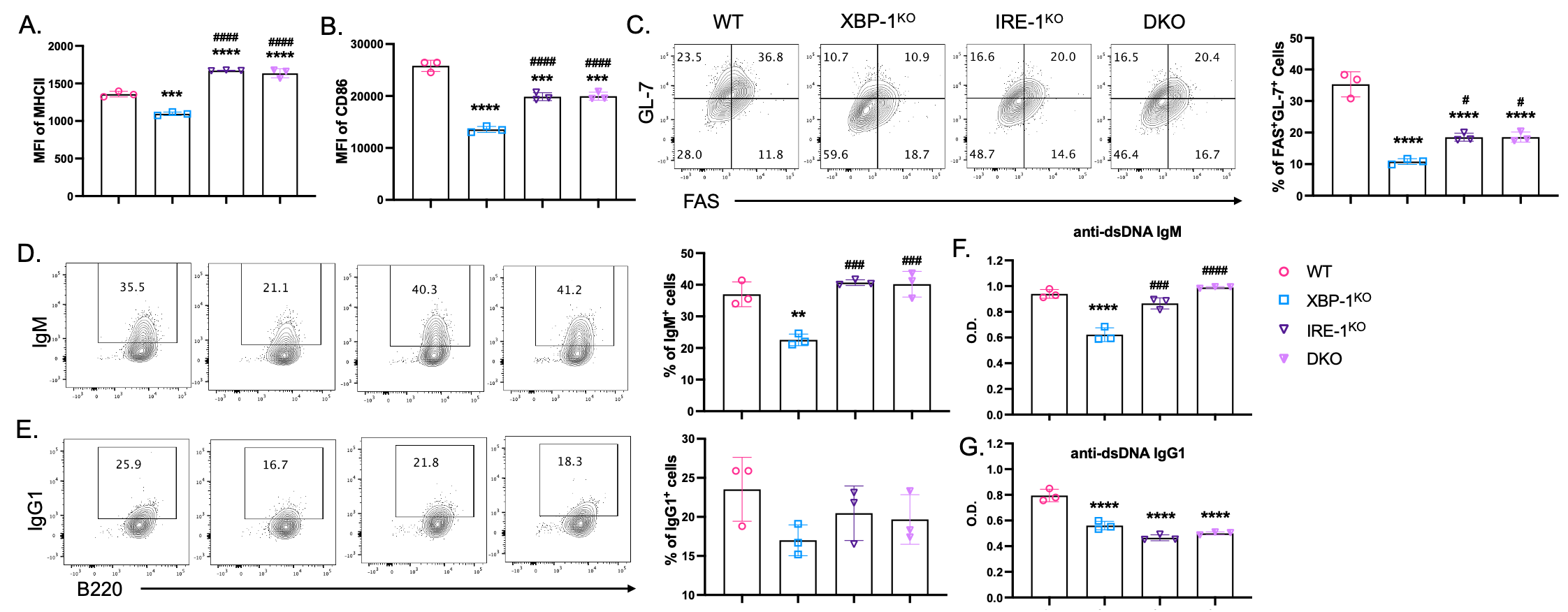
*

**Supplementary Figure 3. Role of XBP-1s and IRE-1α in B cell activation, differentiation, and IgM production after BCR activation.** B cells were isolated from WT, XBP-1KO, IRE-1αKO, and DKO mice and stimulated with F(ab’)2 (10 𝜇g/ml) and anti-CD40 (10 𝜇g/ml) for 48 hrs. Cell surface expression of MHCII (A) and CD86 (B) were measured by flow cytometry analysis. Germinal Center B cells (FAS+GL-7+) were detected by flow cytometry (C). B cells were stimulated with PMA and Ionomycin for another 4 hrs. B cells were intracellularly stained and analyzed for IgM (D) and IgG1 (E) production. Cell supernatants were collected, and anti-dsDNA autoantibodies were detected using ELISA (F and G). Data are shown as means ± SD. MFI, mean fluorescence intensity. Statistics were performed using ordinary one-way ANOVA with Tukey’s multiple comparison test. * p < 0.05, ** p < 0.005, *** p < 0.0005, and **** p < 0.0001 when compared to WT. # p < 0.05, ## p < 0.005, ### p < 0.0005, and #### p < 0.0001 when compared to XBP-1KO group.


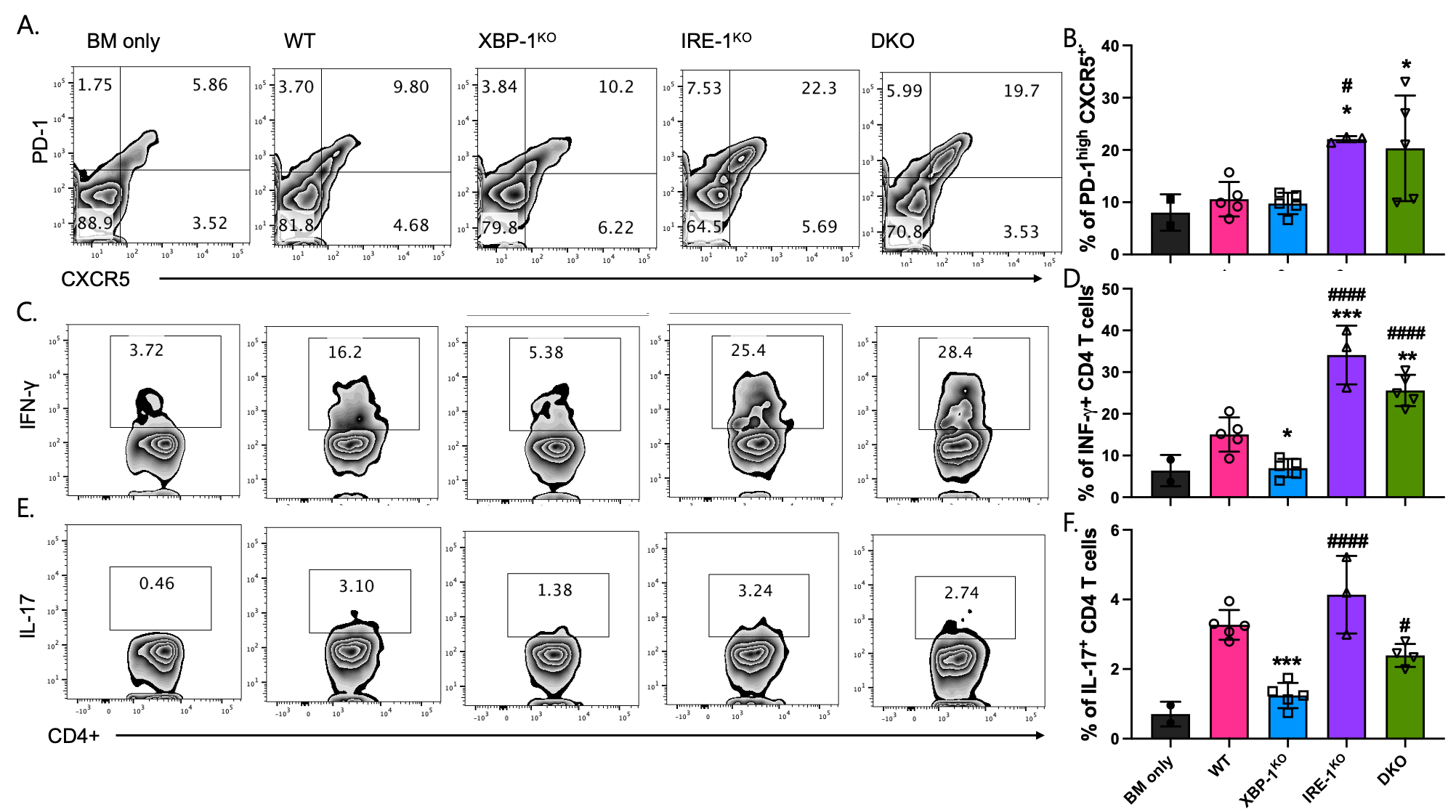


**Supplementary Figure 4. The long-term effect of B cell-specific deficiency of XBP-1s and IRE-1α on T cell activation in a cGVHD model.** BALB/c mice were lethally irradiated and transplanted with 5 x 10^6^ TCD-BM from WT, XBP-1^KO^, IRE-1α^KO^, and DKO mice with or without 0.35 - 0.5 x 10^6^ splenocytes. On day 60 after allo-HCT, recipient mice were euthanized, and single cells were isolated from recipient spleens. (A and B) Donor CD4 follicular T cells (CXCR5^+^PD-1^+^) were analyzed with flow cytometry. Splenocytes were stimulated with PMA and Ionomycin for 4 h and the expression levels of IFN-γ (C and D) and IL-17 (E and F) were determined with intracellular flow cytometry analysis. Statistics were performed using ordinary one-way ANOVA with Tukey’s multiple comparison test. * p < 0.05, ** p < 0.005, *** p < 0.0005, and **** p < 0.0001 when compared to WT. # < 0.05 and #### p < 0.0001 when compared to XBP-1^KO^ group.


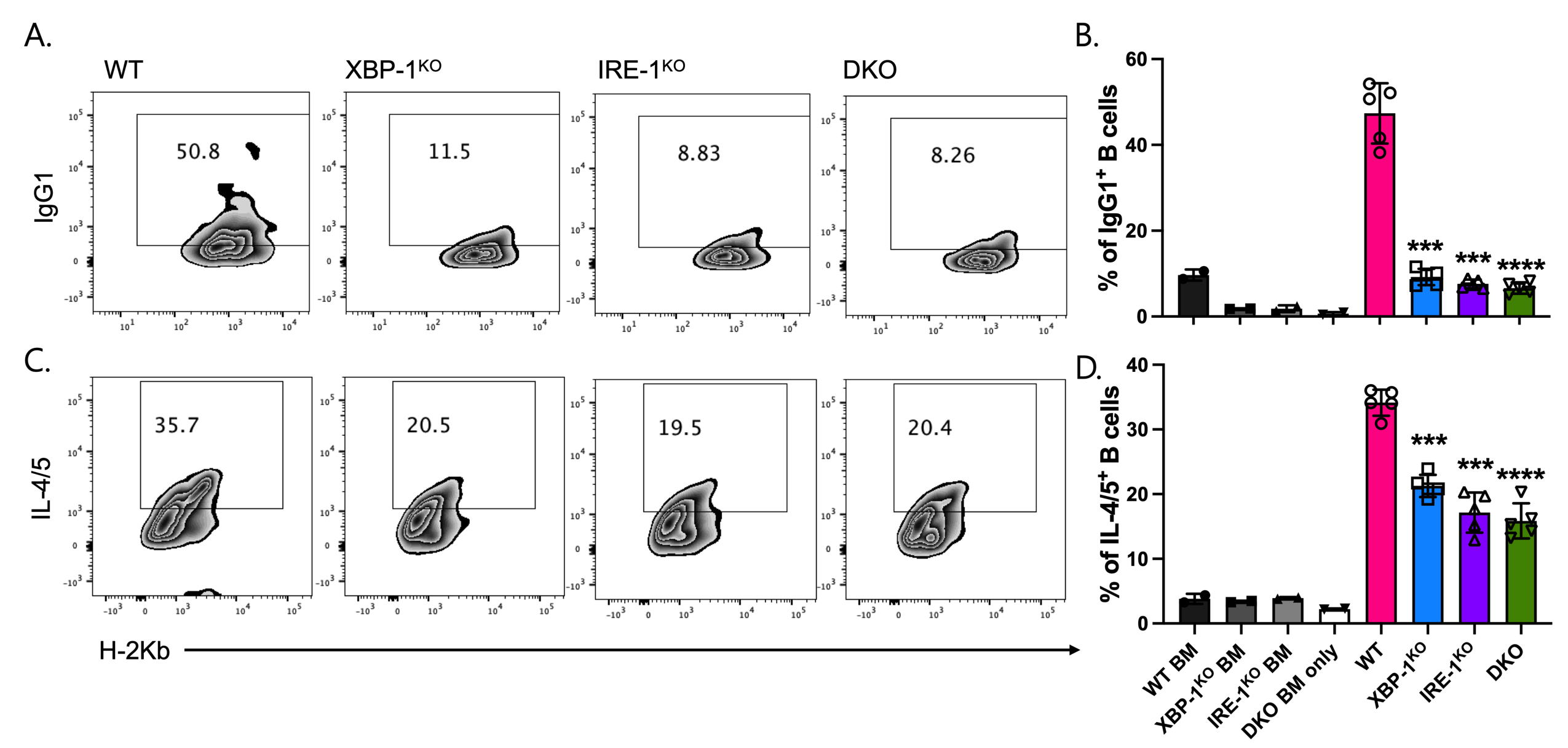


**Supplementary Figure 5. Roles of XBP-1s and IRE-1α in IgG1 and IL-4/5 production by B cells during cGVHD.** BALB/c mice were lethally irradiated and transplanted with 5 x 10^6^ TCD-BM cells from WT, XBP-1^KO^, IRE-1α^KO^, and DKO mice on a B6 background with or without 0.35 - 0.5 x 10^6^ splenocytes. Subsets of recipient mice were euthanized on day 28, and spleens were dissected and processed into single-cell suspensions. Intracellular expression levels of IgG1 (A and B) and IL-4/IL-5 (C and D) were measured by flow cytometry analysis. Statistics were performed using two-way ANOVA with Tukey’s multiple comparison test. **** p < 0.0001 when compared to WT.


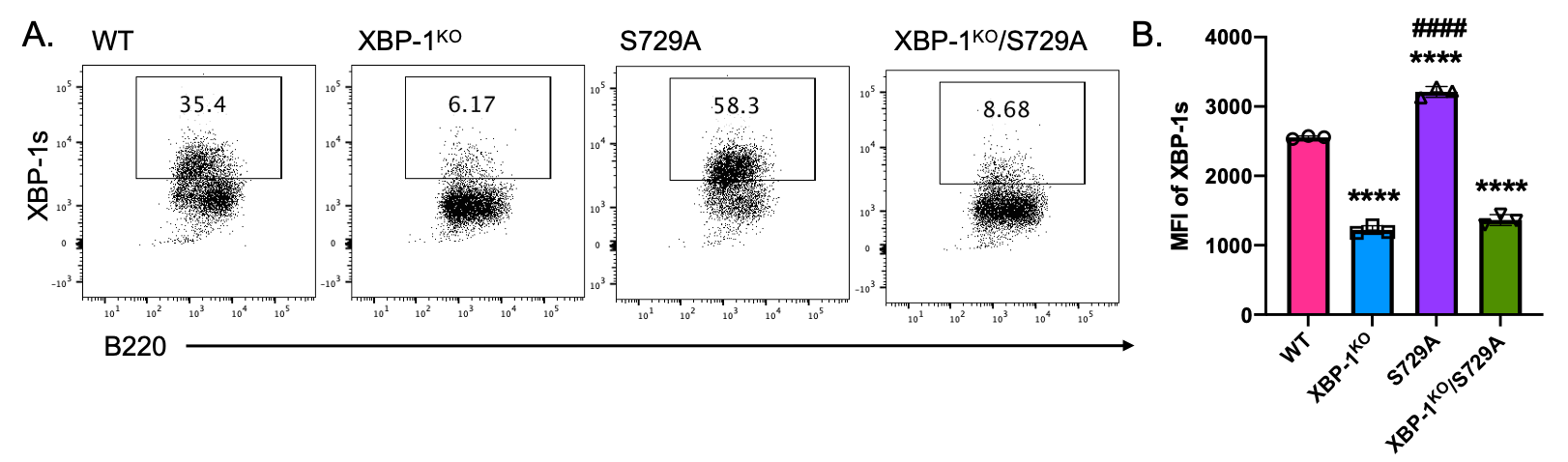


**Supplementary Figure 6. XBP-1s expression levels in WT, XBP-1^KO^, S729A, and XBP-1^KO^/S729A B cells.** WT, XBP-1^KO^, S729A, and XBP-1^KO^/S729A naïve B cells were stimulated with 1 𝜇g/ml LPS and 10 ng/ml IL-4 for 4 days and analyzed for the expression of spliced XBP-1 by flow cytometry. (A) Percentages of XBP-1s- expressing B cells. (B) Mean fluorescence intensity (MFI) of XBP-1s in B cells. Statistics were performed using two-way ANOVA with Tukey’s multiple comparison test. **** p < 0.0001 when compared to WT. #### p < 0.0001 when compared to XBP-1^KO^ group.


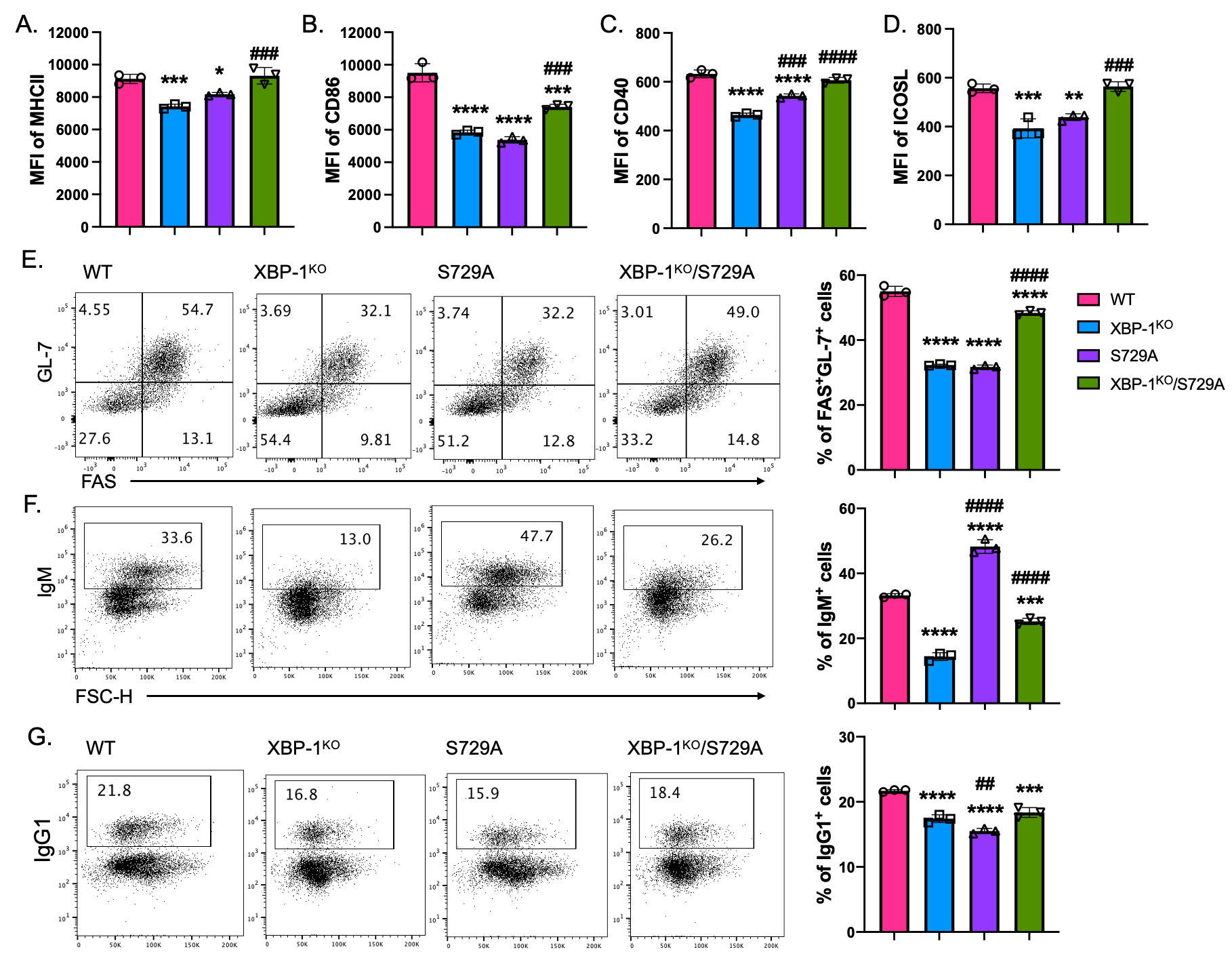


**Supplementary Figure 7. Roles of XBP-1s and the S729A mutation of IRE-1α in B cell activation, differentiation, and IgM production in vitro after LPS and IL-4 stimulation.** B cells were isolated from WT, XBP-1KO, S729A, and XBP-1KO/S729A mice and stimulated with 1 𝜇g/ml LPS and 10 ng/ml IL-4 for 4 days. The expression levels of MHCII (A), CD86 (B), CD40 (C), and ICOSL (D) were detected by flow cytometry. (E) The percentages of germinal center B cells (FAS+GL-7+) were presented. (F-H) B cells were stimulated with PMA and Ionomycin for 4 hrs on day 4. Intracellular levels of IgM (F), and IgG1 (G) were detected by flow cytometry analysis. Data are representative graphs and flow cytometry plots of three repeated experiments. Statistics were performed using two-way ANOVA with Tukey’s multiple comparison test. * p < 0.05, ** p < 0.005, *** p < 0.0005, and **** p < 0.0001 when compared to WT. ### p < 0.0005, and #### p < 0.0001 when compared to XBP-1KO group.


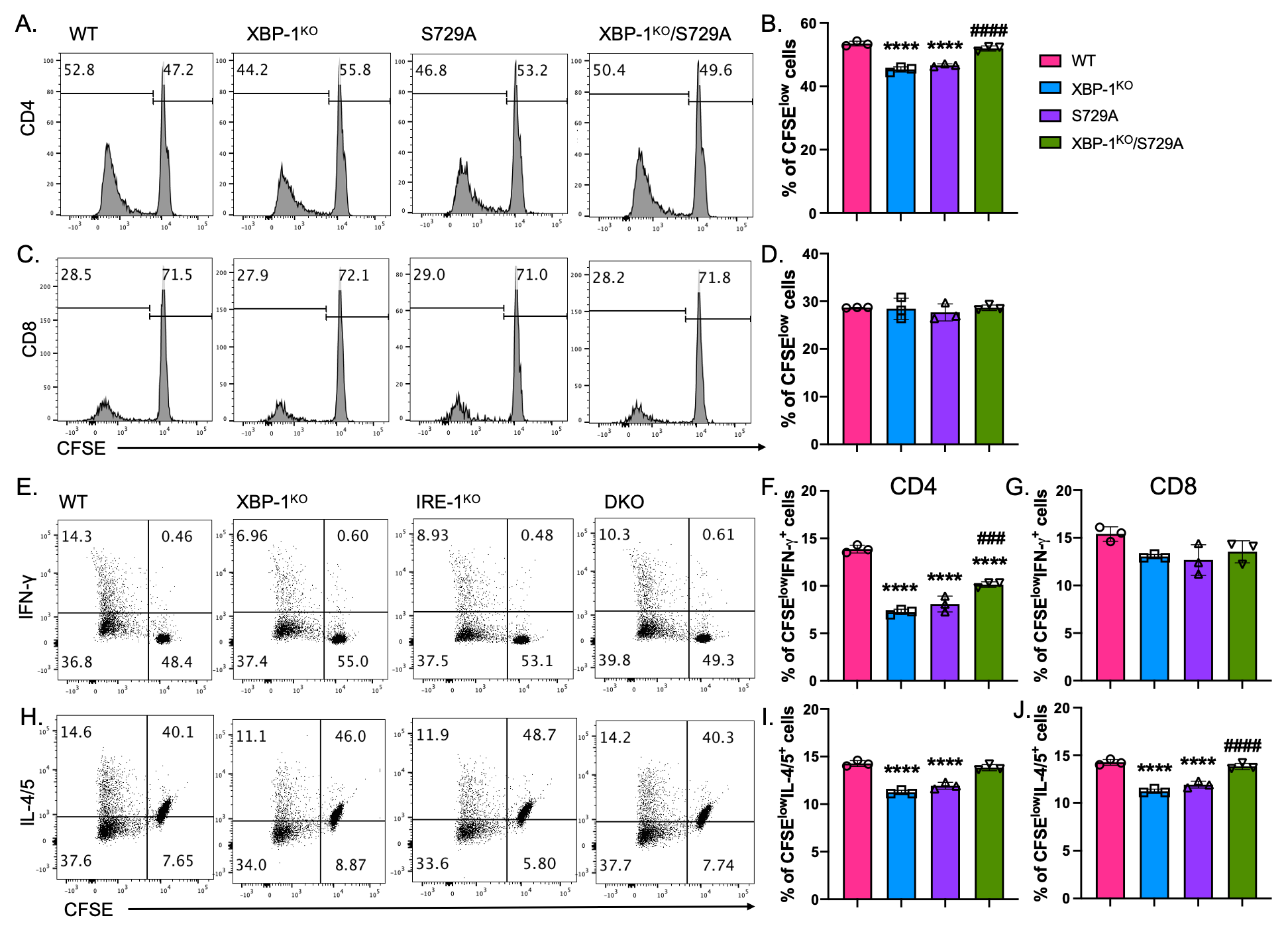


**Supplementary Figure 8. Effects of XBP-1s and the S729A mutation of IRE-1α on B cell ability to stimulate allogeneic T cells in vitro.** B cells from WT, XBP-1KO, S729A, and XBP-1KO/S729A mice were stimulated with 1 𝜇g/ml LPS and 10 ng/ml IL-4 for 24 hrs. CFSE-labeled T cells from FVB mice were added after cytokine removal and incubated for another 4 days. The representative flow panels of CFSE dilution and percentages of CFSE diluted CD4 (A and B) and CD8 (C and D) T cells. T cells were stimulated with PMA and Ionomycin for 4 hrs before intracellular staining for cytokine detection by flow cytometry. The representative flow panels and the percentages of IFN-γ (E-G), and IL-4/5 (H and I) among proliferated (CFSElow) CD4 and CD8 T cells were shown. Data show representative flow cytometry plots and graphs from three repeated experiments. Statistics were performed using two-way ANOVA with Tukey’s multiple comparison test. **** p < 0.0001 when compared to WT. ### p < 0.0005, and #### p < 0.0001 when compared to XBP-1^KO^ group.
